# Adoption of Telehealth Among Older Adults During the COVID-19 Pandemic

**DOI:** 10.1093/geroni/igab046.1649

**Published:** 2021-12-17

**Authors:** Weidi Qin

**Affiliations:** University of Michigan

## Abstract

The COVID-19 pandemic has disrupted older adults’ in-person healthcare services. Many individuals rely on remote communication with their healthcare providers for non-urgent health or mental health issues. The present study investigated the effects of technology learning and depressive symptoms on new adoption of telehealth (e.g. online messaging, video call) to communicate with healthcare providers during the COVID-19 pandemic. A sample of 1,500 Medicare beneficiaries aged 65 or older was selected from the National Health and Aging Trend Study. A series of logistic regressions were performed. Results showed that older adults who learned a new online technology during the COVID-19 outbreak were more likely to adopt telehealth. Also, older adults with a higher level of depressive symptoms were more likely to start using telehealth. The findings highlight the importance of technology training to help older adults go online. Telehealth can be an important coping tool for depressive symptoms during the pandemic.

